# Effect of add-on cathodal transcranial direct current stimulation (c-TDCS) over pre-supplementary motor area (pre-SMA) in patients with obsessive compulsive disorder: A randomized sham controlled study

**DOI:** 10.1192/j.eurpsy.2021.414

**Published:** 2021-08-13

**Authors:** A. Mukherjee, S. Praharaj, S. Rai

**Affiliations:** 1 Department Of Psychiatry, Kasturba Medical College, Manipal, Manipal Academy of Higher Education, Manipal, India; 2 Department Of Clinical Psychology, Manipal College of Health Professions, Manipal Academy of Higher Education, Manipal, India

**Keywords:** ocd, tDCS, brain stimulation, Neuromodulation

## Abstract

**Introduction:**

Patients with OCD often show unsatisfactory response to first-line treatment, giving rise to a need for novel therapeutic approaches. Recent studies using tDCS for OCD treatment have shown promise.

**Objectives:**

To assess efficacy and safety of add-on c-tDCS over pre-SMA compared to sham stimulation in patients with OCD.

**Methods:**

In this double-blinded study, fourteen patients with OCD were randomized to receive 10 sessions of either active (Cathode over pre-SMA, anode over right deltoid, 2mA, 20 minutes per session, 2 sessions per day, 2 hours apart) or sham tDCS. YBOCS, HAM-D, HAM-A, CGI, Wisconsin Card Sorting Test (WCST), and Stroop Test were administered at baseline, post-tDCS, and 1 month post-tDCS.

**Results:**

Group×time interaction effect for YBOCS scores with Repeated Measures ANOVA was not statistically significant, however, reduction in scores in active group was higher, with large effect size (YBOCS scores: Obsessions-η_p_^2^=.344, Compulsions-η_p_^2^= .384, Total-η_p_^2^=.392) (Fig.1 & 2). At 1 month, 42.9% patients in active group and none in sham group showed response. CGI-S score (p=0.016, η_p_^2^=.531) (Fig. 3) and four parameters of WCST (Perseverative responses:p=0.038, η_p_^2^=.448;Percent perseverative responses:p=0.026, η_p_^2^=.485;Percent perseverative errors:p=0.038, η_p_^2^=.447;Trials to complete first category:p=0.011, η_p_^2^=.563) significantly reduced in active group. No significant difference in change in depressive and anxiety symptoms between groups, or change in Stroop Test performance was noted. Adverse effects included transient headache and tingling sensation.

**Figure:**
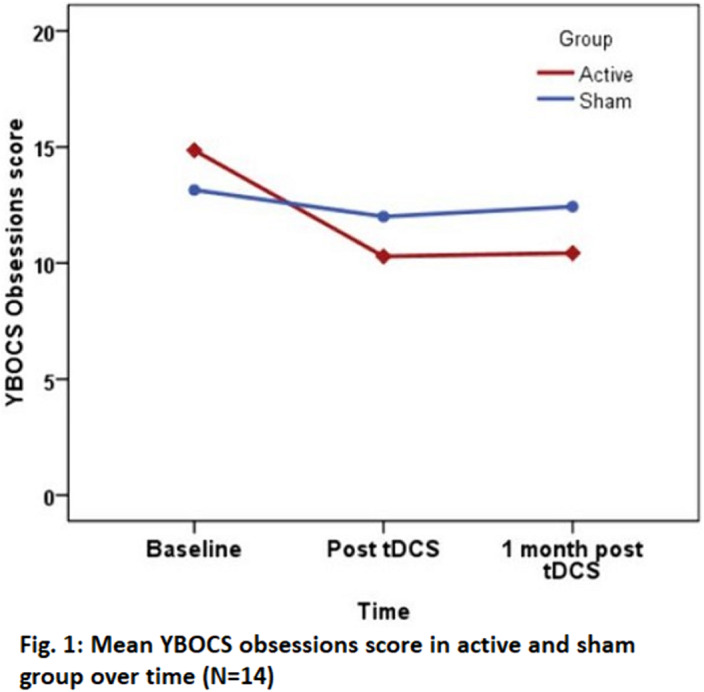

**Figure:**
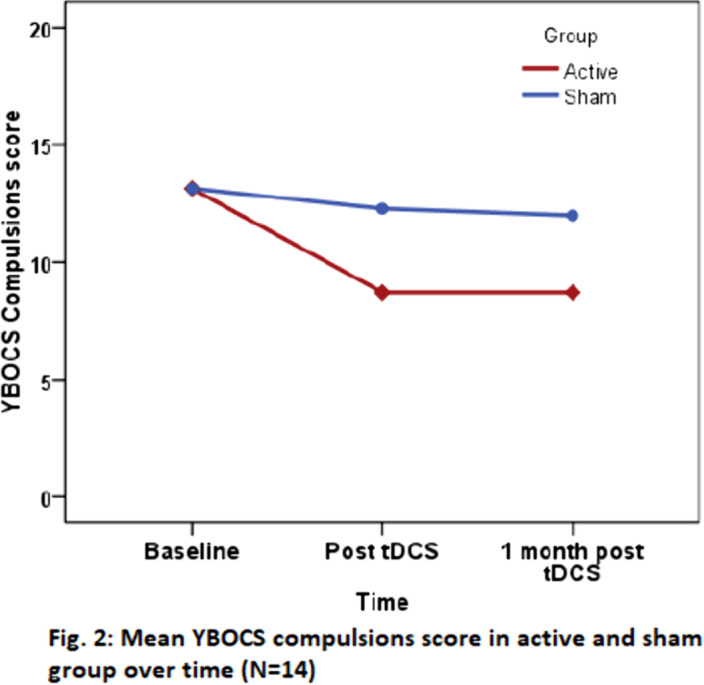

**Figure:**
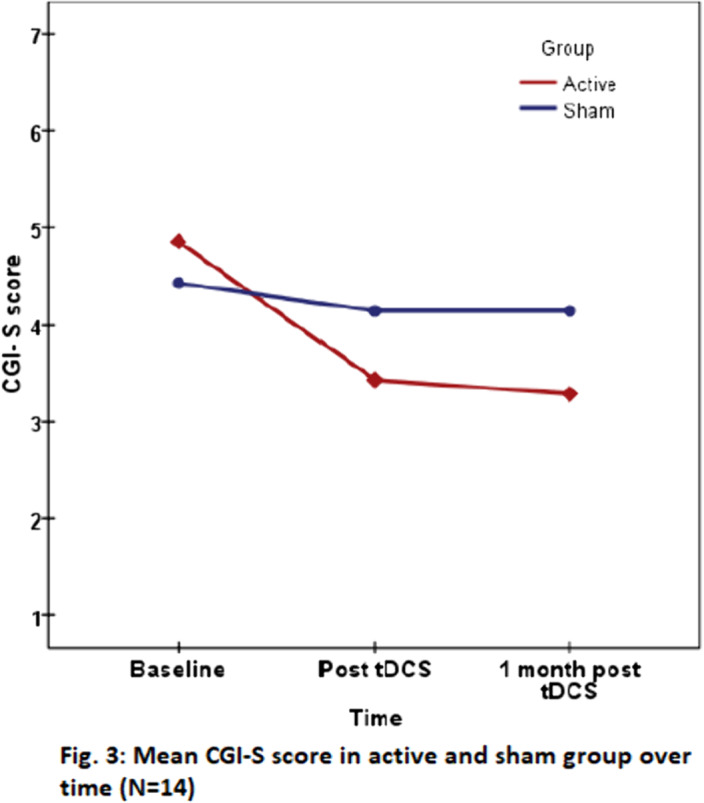

**Conclusions:**

Cathodal tDCS over pre-SMA may be effective in reduction of obsessions, compulsions, illness severity, and enhancing cognitive flexibility in patients with OCD, with no major adverse effects. Larger studies are required to confirm these findings.

**Disclosure:**

No significant relationships.

